# Knowledge and attitude of dental professionals toward COVID-19 in Riyadh, Saudi Arabia: a cross-sectional survey

**DOI:** 10.2478/abm-2021-0034

**Published:** 2021-12-30

**Authors:** Darshan Devang Divakar, Shruti Basavaraj Nimbeni, Abdulaziz A. Al-Kheraif, Aftab Ahmed Khan, Sachin Naik, Sanjeev Balappa Khanagar, Chitra Jhugroo, Basavaraj Nimbeni

**Affiliations:** Department of Dental Health, College of Applied Medical Sciences, King Saud University, Riyadh 11433, Saudi Arabia; Department of Preventive Dentistry, Division of Pediatric Dentistry, College of Dental Sciences, Mustaqbal University, Buraydah, Al-Qassim Province 51431, Saudi Arabia; Dental Public Health, College of Dentistry, King Saud Bin Abdulaziz University for Health Sciences, King Abdulaziz Medical City, National Guard Health Affairs, Riyadh 11426, Saudi Arabia; Agmal Ebtsama Dental and Derma Clinics Center, Buraydah, Al-Qassim Province 51431, Saudi Arabia

**Keywords:** COVID-19, dental, pandemics, preventive health services, SARS-CoV-2

## Abstract

**Background:**

Coronavirus disease-2019 (COVID-19) is a life-threatening global pandemic. The dental profession is considered a high-risk group in the transmission of the responsible virus.

**Objective:**

To assess the knowledge and attitude among dental professionals in response to the COVID-19 pandemic.

**Methods:**

We conducted a cross-sectional study of dental graduates, interns, postgraduates, and dental faculty from May to July 2020. A standardized questionnaire was developed to assess knowledge and attitude of 650 participants. The questionnaire comprised 14 questions to assess general knowledge about COVID-19, 11 questions regarding knowledge about prevention of COVID-19 in dental practice, and 10 questions regarding the attitude toward preventing COVID-19.

**Results:**

Among the study population, only 376 (57.8%) knew the causative virus for COVID-19. Only 425 (65.3%) knew about rinsing the mouth with an antimicrobial solution or 1% hydrogen peroxide before the dental procedure. Regarding the hand hygiene guidelines, 357 (54.9%) had knowledge of the Centers for Disease Control and Prevention (CDC) and 377 (58.0%) about World Health Organization (WHO) guidelines. At the time of our survey, 72% of the participants showed sufficient knowledge, while 28% had low or insufficient knowledge about COVID-19.

**Conclusion:**

While there was a lack of knowledge among dental professionals in Riyadh, Saudi Arabia about COVID-19, there was an excellent positive attitude toward preventing disease. Greater awareness is needed to control the spread of this disease.

A viral pneumonia outbreak originated in Wuhan, China. The causative new coronavirus (CoV), severe acute respiratory syndrome CoV 2 (SARS-CoV-2), causes coronavirus disease-2019 (COVID-19) in humans and has become a pandemic [1]. CoV is zoonotic and causes diseases, including respiratory tract infections, in animals and can be transmitted to humans [2]. The dental care providers are at particular risk as they are exposed to SARS-CoV-2 through the airborne spread, aerosols generated during dental procedures, direct contact with the patient's oral fluids and blood, and contaminated surfaces and instruments during dental procedures [3]. Before the SARS-CoV-2003 global outbreak, only 2 viruses, namely human virus (HCoV-OC43) and HCoV-229E, were known to cause infection in humans. After the outbreak, 5 additional viruses were discovered; including the recently recognized SARS-CoV-2 [4]. CoV were also found to cause the previously reported Middle East respiratory syndrome (MERS) and SARS, which cause life-threatening severe respiratory infections leading to acute respiratory distress syndrome (ARDS), with symptoms like severe shortness of breath and rapid breathing [5]. The most susceptible group is the elderly (>60 years of age). Underlying conditions like diabetes, hypertension, and respiratory diseases may increase the susceptibility for CoV infection. The transmission of SARS-CoV-2 is like other airborne infections and can be through direct contact with infected individuals. The affected individuals can infect multiple people as they can develop high viral burdens and aerosolize them [6].

The current situation because of COVID-19 is worsening, with new cases every day and increasing morbidity as a result. Considering the modes of transmission, health care workers are at high risk of exposure to the virus [7]. Dental care providers need to be very cautious in rendering dental services to their patients. They are expected to evaluate patients appropriately and practice adequate hygiene methods, including the use of personal protective equipment (PPE). Effective disease counteractions and control measures rely upon mindfulness among dental professionals. The Saudi Ministry of Health (MOH) emphasizes strictly following the guidelines of the U.S Centers for Disease Control and Prevention (CDC) and World Health Organization (WHO) for infection control. These guidelines emphasize standard precautions against viral infections, including eye protection to avoid droplets, and masks to avoid airborne aerosols [8]. The present study sought to evaluate the awareness of and mentality toward COVID-19 among dental professionals in a large dental institute in Saudi Arabia. It has addressed inadequate information about the quick spread of the disease in clinics. Strict infection control guidelines are required to prevent airborne and direct contact transmission of SARS-CoV-2 [9]. Hence, the present study aimed to assess the knowledge and attitude of dental professionals in response to the COVID-19 pandemic.

## Methods

### Study design and target population

We conducted a cross-sectional study of dental undergraduates (UG), interns, postgraduates (PG), and faculty of the College of Dentistry, King Saud University, in Riyadh, Saudi Arabia. The College of Dentistry is part of a teaching center comprising 9 departments covering a range of specialties including oral and maxillofacial surgery, oral medicine and diagnostic science, pediatric dentistry and orthodontics, periodontics and community dentistry, prosthetic dental science, and restorative dental science and numbering approximately 80 in faculty. The college has an intake of about 150 undergraduate students per year (for a course of 5 years) and about 40 postgraduate students are admitted per year (for a course of 3 years). Our study was directed by the Strengthening the Reporting of Observational Studies in Epidemiology (STROBE) statement [10]. We had access to only a single university for the study, which was scheduled for 3 months (May–July 2020). We used non-probability convenience sampling to select the participants for study [11]. The study was conducted after approval from the Institutional Review Board of Applied Medical Science King Saud University (No. RP/2020/229/136/135). The UG, interns, PG, and dental faculty in the College of Dentistry, King Saud University were eligible for inclusion in the study, and those who were willing to participate were included.

Sample size was derived at 95% confidence interval through the equation:

$$\mathrm{Sample}\;\mathrm{size}\left(N\right)={\left(Z\right)}^{2}\;p\left(1-p\right)/E^{2}$$

where *Z* = 1.96 (level of confidence 95%), *p* = 50% (assuming 50% participant knowledge), and *E* (Margin of error) = 4%.

The sample size derived was 650 allowing for dropouts. The email addresses and WhatsApp contact numbers of the participants were retrieved from the university. A standardized questionnaire was distributed among all study participants through email and WhatsApp. All participants were informed about the survey and assured that the privacy of the gathered information would be maintained. Documented informed consent was obtained from all participants.

### Questionnaire content

A standardized questionnaire was developed based on the previous studies, and the questions were modified based on the frequently asked questions (FAQ) from the WHO and U.S. Department of Health & Human Services Centers for Disease Control and Prevention (CDC) website [12, 13, 14, 15]. Before being used in the field, the questionnaire content validity was tested among 10 experienced local researchers, dental academics, and health administrators. The panel discussion conducted among the experts for content validity resulted in >0.7 agreement. After content validity, the questionnaire was pilot tested among the 20 participants for face validity, and we found 70% of the participants displayed knowledge about COVID-19. Based on acceptability and face validity, questionnaire modifications were made. We developed 35 questions, and the questionnaire was divided into 4 parts. The first part comprised demographic data like age, sex, nationality, participant details (UG, intern, PG, or faculty). The second part comprised 14 questions regarding general knowledge about COVID-19. The third part comprised 11 questions regarding knowledge about the disease and dental practice. The fourth part comprised 10 questions regarding attitude toward COVID-19.

The scoring system was developed and modified based on similar previous studies [14,15]. To assess the participants’ knowledge, 25 questions were included in the second and third parts of the questionnaire with a Yes or No response. For each correct answer, 1 point was allotted, and no points were deducted or allotted for a wrong answer. Knowledge of participants was divided into 3 categories: low (<15 points), average (16–21 points), and high (>21 points).

Descriptive statistical analysis was conducted to analyze frequency distribution and percentage. Statistical analysis was performed using IBM SPSS Statistics for Windows (version 25).

## Results

Among the 650 dental professionals who participated in the present study, 430 were UG, 100 interns, 50 PG, and 70 were dental faculty (**Table 1**). Among the participants, were 323 (49.7%) men, and 327 (50.3%) were women. Most of the participants received information about COVID-19 from peers (31%) and social media (29%) (**Table 2**). Among the participants, only 376 (57.8%) knew the causative virus for COVID-19. Most of the participants, 632 (97.2%), had knowledge that infection spreads through cough, sneeze, or droplet inhalation through the so called “danger triangle” of the face. Only 252 (38.7%) of the participants responded correctly to the question concerning the mortality rate of SARS-CoV-2 infection; among them, 150 (34.8%) were UG. Among the study participants, 445 (68.4%) participants knew about the incubation period for COVID-19, 598 (92%) were aware that patients with comorbidity (diabetes, hypertension, respiratory disease) are more susceptible to infection, and dental faculty responded with the highest correct response rate 68 (97.1%). Overall, 546 (84.0%) were aware of symptoms of COVID-19, of whom 365 (84.8%) were UG, 467 (71.8%) were aware that there was no vaccination available for COVID-19 at the time of the study, 394 (60.6%) knew that antibiotic prescription was not a first-line treatment for the infection, and only 404 (62.1%) knew that (real-time) reverse transcriptase polymerase chain reaction (RT-PCR) is a standard criterion test for diagnosing COVID-19, 35 (70%) among them being interns. We found that 588 (90.4%) of the participants correctly responded that smokers had increased risk for COVID-19 (**Table 3**). Among the participants, 425 (65.3%) knew that rinsing the mouth with an antimicrobial solution or 1% hydrogen peroxide before the dental procedure mitigates the transmission of infection, and 564 (86.7%) participants agreed dental clinics could promote the transmission of infection. Among the participants, only 357 (54.9%) knew the CDC hand hygiene guidelines, and 377 (58%) were aware of WHO hand hygiene guidelines; 595 (91.5%) knew about using PPE during the treatment, 545 (83.8%) preferred washing hands with soap and water for 30 s or using 60% alcohol-based hand sanitizers, and 513 (78.9%) knew to use N-95 and filtering face-piece (FFP2) masks while treating COVID-19 patients. Fewer participants, 503 (77.3%), responded that a rubber dam could be used for isolation or a high-volume saliva ejector could be used to avoid the aerosols during emergency treatment; 516 (79.3%) participants knew to take extra precautions during aerosol-producing procedures (**Table 4**).

**Table 1:** Distribution of study participants by age and designation

**Sex**	**n (%)**				**Total n (%)**

	**UG**	**Interns**	**PG**	**Faculty**	
Male	200 (46.5)	45 (45)	35 (70)	43 (61.4)	323 (49.7)
Female	230 (53.5)	55 (55)	15 (30)	27 (38.6)	327 (50.3)

Total	430 (66.2)	100 (15.3)	50 (7.6)	70 (10.7)	650 (100)

PG, postgraduates; UG, undergraduates.

**Table 2:** Source of information about COVID-19

**Source**	**Percentage (%)**
MOH	15
Social media	29
Television	17
Peer	31
Newspaper	6
Others	2

COVID-19, Coronavirus disease 2019; MOH, Saudi Ministry of Health.

**Table 3:** Distribution of frequency regarding correct responses to general knowledge about COVID-19

**Question**	**n (%)**				**Total n (%) (n = 650)**

	**UG (n = 430)**	**Interns (n = 100)**	**PG (n** = **50)**	**Faculty (n** = **70)**	
Do you have sufficient knowledge about novel COVID-19?	385 (89.5)	85 (85)	39 (78)	57 (81)	566 (87.0)
Does SARS-CoV-2 cause COVID-19?	280 (65.1)	20 (20)	25 (50)	51 (73)	376 (57.8)
Is COVID-19 zoonotic by origin?	320 (74.5)	57 (57)	35 (70)	40 (57)	452 (69.5)
Is cough, sneeze, and droplet inhalation through danger triangle of the face the main route of transmission for COVID-19 in humans?	421 (97.9)	96 (96)	47 (94)	68 (97)	632 (97.2)
Which of these viral infections can result in high mortality rates – SARS-CoV, MERS-CoV, COVID-19 or HCoV-229E?	150 (34.8)	45 (45)	22 (44)	35 (50)	252 (38.7)
Would it be correct to say that 2 weeks is the median incubation period for COVID-19?	280 (65.1)	60 (60)	44 (88)	61 (87)	445 (68.4)
Would it be correct to say that the elderly are more susceptible for COVID-19?	345 (80.2)	85 (85)	42 (84)	62 (89)	534 (82.1)
Are patients with comorbidity like hypertension, diabetes, respiratory system disease, and cardiovascular diseases at an increased risk for developing COVID-19?	395 (91.8)	90 (90)	45 (90)	68 (97)	598 (92.0)
Do COVID-19 patients develop severe ARDS and cytokine storm?	376 (87.4)	86 (86)	46 (92)	66 (94)	574 (88.3)
Are fever, cough, and shortness of breath main symptoms of COVID-19?	365 (84.8)	75 (75)	43 (86)	63 (90)	546 (84.0)
Is there any vaccination available for COVID-19?	310 (72.0)	65 (65)	32 (64)	60 (86)	467 (71.8)
Can RT-PCR be used for diagnosing COVID-19?	260 (60.4)	54 (54)	35 (70)	55 (79)	404 (62.1)
Can antibiotics be used as the first line of treatment for COVID-19?	230 (53.4)	72 (72)	30 (60)	62 (89)	394 (60.6)
Would it be correct to say that smokers have increased risk of developing COVID-19?	410 (95.3)	84 (84)	39 (78)	55 (79)	588 (90.4)

ARDS, acute respiratory distress syndrome; COVID-19, Coronavirus disease-2019; HCoV, human Coronavirus; MERS, Middle East respiratory syndrome; PG, postgraduates; RT-PCR, reverse transcriptase polymerase chain reaction; SARS-CoV-2, severe acute respiratory syndrome CoV 2; UG, undergraduates.

**Table 4:** Distribution of frequency according to correct responses regarding knowledge about dental practice and COVID-19 transmission

**Questions**	**n (%)**				**Total n(%) (n = 650)**

	**UG (n = 430)**	**Interns (n = 100)**	**PG (n = 50)**	**Faculty (n = 70)**	
Would it be correct to say that special concerns are required to be addressed for patients with recent travel history?	400 (93.0)	85 (85)	42 (84)	60 (86)	587 (90.3)
Can prerinsing patient's mouth with antimicrobial mouthwash or 1% hydrogen peroxide help prevent spread of infection?	300 (69.7)	45 (45)	25 (50)	55 (79)	425 (65.3)
Can patients with COVID-19 spread the infection in dental clinics?	385 (89.5)	75 (75)	39 (78)	65 (93)	564 (86.7)
Are you aware of the U.S. DHHS CDC hand hygiene guidelines?	200 (46.5)	65 (65)	37 (74)	55 (79)	357 (54.9)
Are you aware of the WHO hand hygiene guidelines?	230 (53.4)	63 (63)	32 (64)	52 (74)	377 (58.0)
Do PPE like mouth mask, gloves, gowns, and goggles help prevent the spread of COVID-19?	410 (95.3)	74 (74)	42 (84)	69 (99)	595 (91.5)
Are N-95 or FFP2 masks recommended for dental procedures?	355 (82.5)	67 (67)	35 (70)	56 (80)	513 (78.9)
Can washing hands with soap and water for 30 s or using 60% of alcohol-based hand sanitizers, before and after dental treatment, reduce the transmission of COVID-19 infection?	375 (87.2)	69 (69)	37 (74)	64 (91)	545 (83.8)
Do you use extraoral radiography, panoramic radiography in case of emergency treatment?	360 (83.7)	75 (75)	38 (76)	68 (97)	541 (83.2)
Do you ensure that additional precautions are taken while treating patients with aerosol-generating procedures (ultrasound scaler, 3-way syringe, aerator)?	340 (79.0)	79 (79)	39 (78)	58 (83)	516 (79.3)
Do you use rubber dam for isolation or high-volume saliva ejector in case of emergency treatment?	335 (77.9)	65 (65)	42 (84)	61 (87)	503 (77.3)

CDC, Center for Disease Control and Prevention; COVID-19, Coronavirus disease-2019; DHSS, Department of Health & Human Services; FFP2, filtering face-piece; PPE, personal protective equipment; PG, postgraduates; UG, undergraduates; U.S., United States of America; WHO, World Health Organization.

**Figure 1:**
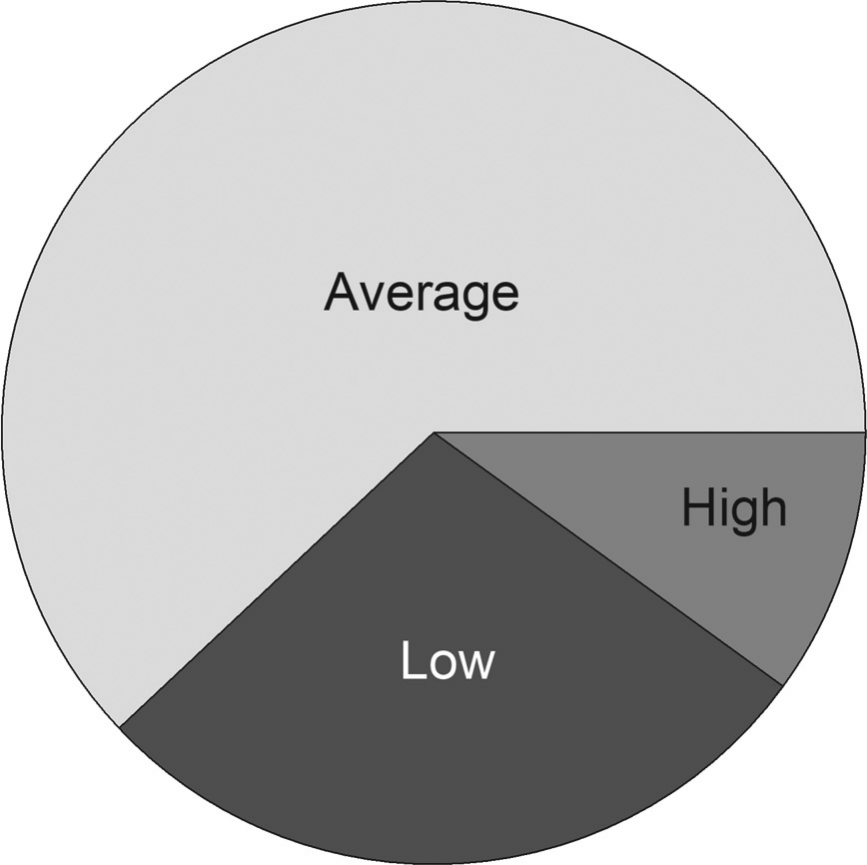
Knowledge about COVID-19 among study participants based on a scoring system. The scoring system was developed and modified based on similar previous studies [14, 15]. To assess the participants’ knowledge, 25 questions were included in the second and third parts of the questionnaire with a Yes or No response. For each correct answer, 1 point was allotted, and no points were deducted for the wrong answer. Knowledge of participants is divided into 3 categories: low (<15 points), average (16–21 points), and high (>21 points). COVID-19, Coronavirus disease.

Based on the scoring system among 650 study participants, 182 (28%) scored low, 403 (62%) average, and 65 (10%) scored high. Thus, 468 (72%) participants showed sufficient knowledge about the COVID-19 (**Figure 1**).

We found an excellent positive attitude response among dental graduates and faculty regarding the prevention of COVID-19. Among study participants, 613 (94.3%) believed in disseminating information regarding COVID-19 among peers and other dental health care workers, and 617 (94.9%) participants believed sterilization of clinical and nonclinical areas in the dental clinic reduces the transmission of infection. We found 588 (90.4%) participants believe that there is an association between hand hygiene and the spread of COVID-19 infection; among them, 395 (91.8%) were UG and 88 (88%) interns (**Table 5**).

**Table 5:** Distribution of frequency of correct responses to questions concerning attitude regarding COVID-19 transmission in dental practice

**Questions**	**n (%)**				**Total n(%) (n = 650)**

	**UG (n = 430)**	**Interns (n = 100)**	**PG (n = 50)**	**Faculty (n = 70)**	
Should dental health workers inform themselves with information regarding COVID-19?	405 (94.1)	85 (85)	45 (90)	70 (100)	605 (93.0)
Do you agree that information regarding COVID-19 should be disseminated among your peers and other dental health care workers?	398 (92.5)	97 (97)	48 (96)	70 (100)	613 (94.3)
Do you agree that an asymptomatic patient is not infected by COVID-19?	365 (84.8)	75 (75)	43 (86)	64 (91)	547 (84.1)
Do you suggest patients to approach a general physician rather than trusting online sources and social media?	386 (89.7)	89 (89)	45 (90)	61 (87)	581 (89.3)
Do you consider that patients with COVID-19 symptoms must be kept in isolation?	370 (80.0)	78 (78)	43 (86)	64 (91)	555 (85.3)
Do you notify dental patient receiving and diagnosis area personnel regarding COVID-19?	400 (93.0)	89 (89)	42 (84)	65 (93)	596 (91.6)
Will you instruct the dental assistants to adopt PPE and proper hand hygiene methods while performing dental procedures?	410 (95.3)	89 (89)	38 (76)	67 (96)	604 (92.9)
Do you believe sterilization of clinical and nonclinical areas can prevent COVID-19 transmission in dental clinics?	415 (96.5)	92 (92)	45 (90)	65 (93)	617 (94.9)
Do you believe that there is an association between hand hygiene and COVID-19 infection control?	395 (91.9)	88 (88)	42 (84)	63 (90)	588 (90.4)
Do you think hand hygiene prevents the spread of COVID-19 infection to patients?	412 (95.8)	90 (90)	49 (98)	66 (94)	617 (94.9)

COVID-19, Coronavirus disease-2019; PPE, personal protective equipment; PG, postgraduates; UG, undergraduates.

## Discussion

Because the COVID-19 pandemic is spreading rapidly, health care professionals need to understand the pathology of the disease to protect themselves from the virus and prevent the spread of the virus. Dental professionals need to know about the disease, as they are a high-risk group for infection, and dentists can educate the patients about personal preventive measures. According to the study participants, the majority (31%) received information about COVID-19 primarily from peer groups, and secondly from web-based social networking. Social media has been the leading facilitator of data broadcasting. In the medical services framework, data broadcasting is crucial to the early dissemination of information [16]. By contrast, social media can be disastrous by spreading false news regarding the disease. Considering this, people should approach general physicians or the MOH for information regarding the disease.

We found that substantially few (57.8%) participants knew that SARS-CoV-2 was the causative agent for COVID-19. This lack of knowledge may be because, during new episodes of infection, there is a lack of information among the participants about the name of the infective agent and perhaps because the disease name is not the same as the name of the infection [17]. In the present study, most participants (97.2%) knew that the COVID-19 infection spread by coughing, sneezing, and droplet inhalation. Gravity settles the heavy droplets, but aerosols produced by a dental procedure can spread to long distances. Using ultrasonic instruments and aerators can produce aerosols in substantial amounts [18].

Along with cough, sneezing, fever symptoms accompanied by ARDS are the leading and life-threatening COVID-19. During infection, the organ-protective effect of angiotensin-converting enzyme 2 and an increase in cytokine levels can lead to a so-called “cytokine storm” in the immune system [19]. It is essential for dental professionals to understand the pathophysiology, diagnosis, and differential diagnosis of the disease.

The mortality rates for COVID-19 in China were estimated at 5.6%, and outside China, it was estimated to be 15.2%; and the mortality rate of MERS was at the highest, ranging from 6% to 28% [20, 21]. Only 38.7% of our participants were aware of the mortality rate of COVID-19. This may be because COVID-19 has infected a sizable proportion of the population, and biased information about the disease's mortality rate has spread. COVID-19 has a high mortality rate in the elderly [22]. The incubation period of COVID-19 is a challenge for control; most patients become symptomatic within 11 days or 12 days, and most of them within 14 days [23]. A similar study among dental professionals reported 46.1% of the respondents as having knowledge about the incubation period, compared with the 68.4% in our study [24]. A dental professional should know that an asymptomatic patient may be in the incubation period and spread the infection. It is recommended to suspect every patient as infectious [25].

In the present study, most participants knew that patients with comorbidity are more susceptible to COVID-19, and that their prognosis will be poor. This might be because most dental experts are aware that comorbidities, like hypertension, diabetes, and respiratory ailments, can lead to susceptibility to many illnesses [26]. RT-PCR is the criterion standard test used for the etiological diagnosis of COVID-19 on respiratory tract specimens, and awareness of this test was found in substantially fewer of the study participants [27].

In the present study, 60.6% knew that antibiotics are not the first line of treatment, and that the empirical use of antibiotics recommended by WHO to reduce bacterial superinfections would not help to treat this disease [28]. To the date the study was conducted, few vaccines were available for COVID-19, SARS-CoV, and MERS. Awareness programs or online information are needed to create awareness about the vaccine and first-line treatment protocol among dental professionals [29].

In the present study, most participants (90.4%) agreed that smoking would increase the susceptibility to COVID-19. To date, there are limited data available about smoking and COVID-19. However, it is being assumed that smoking is associated with a negative impact on lung health. There is evidence that a larger proportion of smokers with COVID-19 were in intensive care units than other patients who were nonsmokers; and moreover, symptoms were more severe in smokers. A previous review study suggests that smoking will worsen the progression and adverse outcomes associated with COVID-19. Smokers are 2.4 times more likely to be admitted to an intensive care unit, need mechanical ventilation, or die compared with nonsmokers [30, 31]; patients with smoking and influenza would have the worst disease prognosis. Smoking patients with MERS had a high mortality [32].

Dental clinics invariably have a high risk for transmission of COVID-19 infection because of the specific treatment methods used, which involve face-to-face interaction with patients, frequent exposure to blood, saliva, and other body fluids, and the use of sharp instruments. The pathogenic virus can be transferred from one patient to another in dental settings through breathing of airborne microorganisms that remain present in the air for an extended period [17]. Aerosol-producing procedures like ultrasonic scalers, aerator, and using a 3-way syringe may spread the infection. Aerosols may infect over long distances. Using a rubber dam is highly recommended [3]. In our study, 86.7% of the participants agreed dental clinics could transmit infection by the aerosols produced and contact spread.

Before beginning a dental treatment procedure, it is advisable to use an antimicrobial mouthwash to rinse the patient's mouth. This decreases the viral load in saliva and avoids spatter. Because SARS-CoV-2 is susceptible to oxidation, the use of a mouth rinse, such as one containing 1% hydrogen peroxide or 0.2% povidone is recommended [20]. In the present study, 65.3% of the study population knew that rinsing the mouth with the antimicrobial solution before the commencing dental treatment could help prevent the spread of infection.

Among the study population, only 54.9% knew about CDC hand hygiene guidelines, and 58% were aware of WHO hand hygiene guidelines; 83.3% preferred washing hands with soap and water for 30 s or using 60% of alcohol-based hand sanitizers. There is evidence that hand hygiene can reduce respiratory infections.

Using a mouth mask and proper hand hygiene reduces influenza transmission successfully [33]. Dentistry is a profession that needs to adopt evidence-based scientific procedures to avoid transmission [34]. As per the CDC and WHO guidelines, comparatively fewer participants knew about the scientific procedure of washing hands before and after examining the patients [35]. This may be the result of a lack of awareness programs among dental professionals.

Most of the study participants (91.5%) knew about using PPE during the treatment. Wearing PPE, such as masks, gloves, eyeglasses, and gowns can help avoid most infections, and most of the study participants had good knowledge about PPE [3], and that N-95 or FFP2 masks are recommended to avoid transmission of virus particles [36].

We found a positive attitude response among dental graduates and faculty regarding the prevention of COVID-19. Among study participants, 94.3% believed in disseminating information regarding COVID-19 among peers and other dental health care workers. Disseminating the information among dental health workers without delay is recommended. The attitude among study participants is similar to that found in an earlier study conducted about the attitude toward MERS [14].

Among the study participants, 94.9% believed in sterilizing clinical and nonclinical areas in the dental clinic helps prevent the infection. The results showed that 90.4% of participants believed that there is an association between hand hygiene and the spread of COVID-19 infection. The lack of a scientific approach toward hand hygiene was recorded among study participants. This shows that the study participants had a positive attitude toward preventing the disease.

A limitation of the present study is that it was restricted to only one institution, and social desirability bias may affect the results by over-reporting good knowledge and attitude. Further similar studies need to be conducted in Saudi Arabia to assess knowledge and attitude among dental professionals and are warranted.

## Conclusions

We conclude that there was a lack of knowledge about the COVID-19 pandemic and COVID-19 infection preventive measures among dental students and faculty, and that greater awareness is needed to control the spread of this disease. We have provided an insight into COVID-19, and suggestions to be followed by the public, governments, and the health care sector to control the spread of COVID-19 infection.

## References

[1] Zhu N, Zhang D, Wang W, Li X, Yang B, Song J (2020). A novel coronavirus from patients with pneumonia in China. N Engl J Med.

[2] Perlman S, Netland J (2009). Coronaviruses post-SARS: update on replication and pathogenesis. Nat Rev Microbiol.

[3] Peng X, Xu X, Li Y, Cheng L, Zhou X, Ren B (2020). Transmission routes of 2019-nCoV and controls in dental practice. Int J Oral Sci..

[4] Giwa A, Desai A (2020). Novel coronavirus COVID-19: an overview for emergency clinicians. Emerg Med Pract..

[5] Fung S-Y, Yuen K-S, Ye Z-W, Chan C-P, Jin D-Y (2020). A tug-of-war between severe acute respiratory syndrome coronavirus 2 and host antiviral defence: lessons from other pathogenic viruses. Emerg Microbes Infect.

[6] Guo Y-R, Cao Q-D, Hong Z-S, Tan Y-Y, Chen S-D, Jin H-J (2020). The origin, transmission and clinical therapies on coronavirus disease 2019 (COVID-19) outbreak – an update on the status. Mil Med Res..

[7] Ran L, Chen X, Wang Y, Wu W, Zhang L, Tan X (2020). Risk factors of healthcare workers with corona virus disease 2019: a retrospective cohort study in a designated hospital of Wuhan in China. Clin Infect Dis.

[8] Baseer M-A, Ansari S-H, AlShamrani S-S, Alakras A-R, Mahrous R, Alenazi A-M (2016). Awareness of droplet and airborne isolation precautions among dental health professionals during the outbreak of corona virus infection in Riyadh city, Saudi Arabia. J Clin Exp Dent..

[9] Kanaparthi A, Dukkireddy D, Gopalaiah H, Kesary SPR, Katne T, Gantala R (2020). Awareness of COVID 19 pandemic among dental practioners of Telangana state, India: a cross sectional survey. J Oral Biol Craniofacial Res.

[10] von Elm E, Altman DG, Egger M, Pocock SJ, Gotzsche PC, Vandenbroucke JP, STROBE Initiative (2008). The Strengthening the Reporting of Observational Studies in Epidemiology (STROBE) Statement: guidelines for reporting observational studies. J Clin Epidemiol.

[11] Burns KEA, Duffett M, Kho ME, Meade MO, Adhikari NKJ, Sinuff T, Cook DJ, for the ACCADEMY Group (2008). A guide for the design and conduct of self-administered surveys of clinicians. CMAJ.

[12] World Health Organization (2020). Coronavirus disease (COVID-19) [Internet].

[13] U.S. Department of Health & Human Services Centers for Disease Control and Prevention Infection control guidance for healthcare professionals about coronavirus (COVID-19) [Internet].

[14] Althomairy S, Baseer M, Assery M, Alsaffan A (2018). Knowledge and attitude of dental health professionals about Middle East respiratory syndrome in Saudi Arabia. J Int Soc Prev Community Dent.

[15] Almutairi KM, Al Helih EM, Moussa M, Boshaiqah AE, Saleh Alajilan A, Vinluan JM (2015). Awareness, attitudes, and practices related to coronavirus pandemic among public in Saudi Arabia. Fam Community Health.

[16] International Civil Aviation Organization Preventing spread of coronavirus disease 2019 (COVID-19) guideline for airports [Internet].

[17] Lauer SA, Grantz KH, Bi Q, Jones FK, Zheng Q, Meredith HR (2020). The incubation period of coronavirus disease 2019 (covid-19) from publicly reported confirmed cases: estimation and application. Ann Intern Med.

[18] Smieszek T, Lazzari G, Salathé M (2019). Assessing the dynamics and control of droplet- and aerosol-transmitted influenza using an indoor positioning system. Sci Rep..

[19] Pedersen SF, Ho Y-C (2020). SARS-CoV-2: a storm is raging. J Clin Invest.

[20] Lapointe L, Ramaprasad J, Vedel I (2014). Creating health awareness: a social media enabled collaboration. Health Technol (Berl).

[21] Alfaraj SH, Al-Tawfiq JA, Assiri AY, Alzahrani NA, Alanazi AA, Memish ZA (2019). Clinical predictors of mortality of Middle East respiratory syndrome coronavirus (MERS-CoV) infection: a cohort study. Travel Med Infect Dis.

[22] Onder G, Rezza G, Brusaferro S (2020). Case-fatality rate and characteristics of patients dying in relation to COVID-19 in Italy. JAMA.

[23] Baud D, Qi X, Nielsen-Saines K, Musso D, Pomar L, Favre G (2020). Real estimates of mortality following COVID-19 infection. Lancet Infect Dis.

[24] Alwazzan RA, Baseer MA, ALMugeiren OM, Ingle NA (2021). Dental professional's knowledge, preventive awareness and attitude towards COVID-19 in Saudi Arabia: a cross-sectional survey. Risk Manag Healthc Policy.

[25] Ather A, Patel B, Ruparel NB, Diogenes A, Hargreaves KM (2020). Coronavirus disease 19 (COVID-19): implications for clinical dental care. J. Endod.

[26] Guan W-j, Liang W-h, Zhao Y, Liang H-r, Chen Z-s, Li Y-m (2020). Comorbidity and its impact on 1590 patients with Covid-19 in China: a nationwide analysis. Eur Respir J..

[27] Lippi G, Simundic A-M, Plebani M (2020). Potential preanalytical and analytical vulnerabilities in the laboratory diagnosis of coronavirus disease 2019 (COVID-19). Clin Chem Lab Med.

[28] World Health Organization (2021). COVID-19 clinical management: living guidance, 2nd edition [Internet].

[29] Ralph R, Lew J, Zeng T, Francis M, Xue B, Roux M (2020). 2019-nCoV (Wuhan virus), a novel coronavirus: human-to-human transmission, travel-related cases, and vaccine readiness. J Infect Dev Ctries.

[30] Hopkinson NS, Rossi N, El-Sayed Moustafa J, Laverty AA, Quint JK, Freidin M (2020). Current smoking and COVID-19 risk: results from a population symptom app in over 2.4 million people. Thorax.

[31] Vardavas CI, Nikitara K (2020). COVID-19 and smoking: a systematic review of the evidence. Tob Induc Dis..

[32] Tonnesen P, Marott JL, Nordestgaard B, Bojesen SE, Lange P (2019). Secular trends in smoking in relation to prevalent and incident smoking-related disease: a prospective population-based study. Tob Induc Dis..

[33] Warren-Gash C, Fragaszy E, Hayward AC (2013). Hand hygiene to reduce community transmission of influenza and acute respiratory tract infection: a systematic review [Internet]. Influenza Other Respir Viruses.

[34] McGlone P, Watt R, Sheiham A (2001). Evidence-based dentistry: an overview of the challenges in changing professional practice. Br Dent J.

[35] Khanagar S, Kumar A, Naik S, Neelakantappa H, Ramachandra S, Vadavadagi S (2014). Knowledge, attitude, and practice of hand hygiene among dentists practicing in Bangalore city – a cross-sectional survey. J Int Soc Prev Community Dent.

[36] Wiwanitkit V (2006). N-95 face mask for prevention of bird flu virus: an appraisal of nanostructure and implication for infectious control. Lung.

